# Altruists Proliferate Even at a Selective Disadvantage within Their Own Niche

**DOI:** 10.1371/journal.pone.0128654

**Published:** 2015-06-01

**Authors:** Bryan Wilder, Kenneth O. Stanley

**Affiliations:** Department of Electrical Engineering and Computer Science, University of Central Florida, Orlando, FL, USA; University of Massachusetts, UNITED STATES

## Abstract

The evolutionary origin of altruism is a long-standing puzzle. Numerous explanations have been proposed, most prominently based on inclusive fitness or group selection. One possibility that has not yet been considered is that new niches will be created disproportionately often when altruism appears, perhaps by chance, causing altruists to be over-represented in such new niches. This effect is a novel variant of group selection in which altruistic groups benefit by discovering unoccupied niches instead of by competing for the limited resources within a single niche. Both an analytical population genetics model and computational simulations support that altruism systematically arises due to this side effect of increased carrying capacity even when it is strongly selected against within any given niche. In fact, even when selection is very strongly negative and altruism does not develop in most populations, it can still be expected to be observed in a consistent fraction of species. The ecological structure provided by niches thereby may be sufficient for altruists to proliferate even if they are always at a disadvantage within each niche considered individually.

## Introduction

Explaining how altruism can evolve is a long-standing challenge. Existing theories fall largely into a three key categories [1]. Inclusive fitness hypothesizes that altruism is favored when the benefit to the individual’s relatives outweighs the cost to the individual [2–5]. Group selection theories argue that an altruist’s actions increase its group’s reproductive success even at a cost to the individual [6–8]. Finally, reciprocity-based theories claim that altruism eventually leads other individuals to cooperate with the altruist, either directly [9, 10] or indirectly [11–13].

This paper introduces a novel form of group selection. Group selection theories fall roughly into two categories [14, 15]. One class of models, associated with early works by Wright, Maynard-Smith, and others [16–19], assumes that the overall population is partitioned into distinct groups with little movement between them. Altruists help their own group survive, and potentially recolonize the space left empty by extinct groups. In these models, there is a distinct process of selection at the level of the group. The second category, often called *intrademic selection*, encompasses more porous groups [6, 20, 21]. In this view, different sets of individuals interact with different frequencies, but contribute to the same reproductive pool. For example, organisms may interact mostly with a small group during some point of their life cycle. This variation in interaction allows selection to act on multiple levels even though individuals are not partitioned into entirely separate groups. A common thread between both of these types of theories is that evolution takes place in a fixed ecology. That is, populations all exist in a single ecological space, competing for the same set of resources. Altruists help their group secure these resources, which increases the chance of reproductive success for all of the group’s members.

In general, though, these conditions do not necessarily hold in nature because of the availability of many distinct ecological niches. While the term “niche” is difficult to define precisely, it corresponds to the role that an organism plays in its ecosystem, defined by the resources that it consumes and produces, or the habitat that it occupies [22]. In addition to competing more effectively against groups within the same niche, organisms also evolve to fill *new* niches with the benefit of less competition [23–25] Moreover, the set of niches can itself change over the course of evolution [26–28]. The space of possible niches depends on what kinds of behaviors are already present, with each innovation opening up opportunities for new developments. Groups can reproduce more effectively not just by increasing their competitive advantage relative to others, but also by increasing their ability to exploit new niches as they become available.

This paper focuses on the insight altruism could facilitate this process. Altruists in this view pay a cost to their own fitness to support another member of their group. The implication is that groups with a larger number of altruists are able to support individuals who would not otherwise survive. These genetic paths, which would otherwise be unexplored, represent additional opportunities for future mutations to find new niches. Real fitness landscapes are often rough due to factors such as epistasis [29–31], so beneficial behaviors may not be discovered if populations move strictly in the direction of greatest fitness. In effect, allowing only the fittest individuals to survive easily traps the population at local optima. When altruists allow a greater diversity of individuals to survive and reproduce, they increase the likelihood that some member of their group will found a new niche. The implication is that the descendants of groups with a higher concentration of altruists will be quicker to fill newly available niches, and as a consequence, the proportion of altruists in future generations will increase.

These considerations suggest that models that incorporate a broader evolutionary process could reveal new mechanisms by which group selection can operate. Altruists might proliferate not just by helping their group compete with others, but also by opening up new possibilities altogether. The experiments in this paper demonstrate the theoretical feasibility of this hypothesis through an analysis of population dynamics in the presence of multiple niches.

## Materials and Methods

We begin by considering an extension to the Wright-Fisher model of population genetics, which has been extensively studied [32], and is a classical model for generational evolution. Suppose there are two alleles, representing altruists and nonaltruists. Altruist pay a fitness cost *s*, which is the excess fitness of nonaltruists relative to alruists. While fitness arises from random interactions between individuals, the Wright-Fisher model abstracts away these interactions and thereby fixes a fitness value that depends only on type. Typically, the population size *N* is fixed. However, to model the potential for altruism to increase carrying capacity in general, we allow *N* to vary linearly between *N*_base_ and *βN*_base_ according to the fraction of altruists in the population. Here, *β* is a parameter that controls the effect of altruism on carrying capacity. The experiments focus on *β* > 1 to explore the case where altruists increase carrying capacity; clearly altruism will not survive if it is detrimental to both the individual and the group. The mathematical details of the model are given in S1 Text. It is similar to the one proposed by Houchmandzadeh [33] but also includes mutation with probability *μ* to study populations that randomly fluctuate without reaching fixation. The strength of selection is given by the product *Ns*. Because *N* is not constant in this model, $N_{\text{mid}}=\frac{N_{\text{base}}+\beta N_{\text{base}}}{2}$ stands in for *N*. A weak selection regime is then given by *N*_mid_ *s* ≪ 1, and strong selection by *N*_mid_ *s* ≫ 1. The dynamics of the model form an ergodic Markov chain. Across a range of parameter settings, the probability can be obtained that some fraction of individuals are altruists in the stationary distribution. These probabilities are found explicitly by solving a system of linear equations given by the transition matrix of the chain. The novel consideration is the possibility of mutation to a new evolutionary niche. Such niche-founding occurs with some small probability *μ*_ev_ for each new offspring. This analytic model does not by design track the evolutionary dynamics of new niches created by these mutations. Instead, the focus is on the composition of the population at the stationary distribution compared to the time when a mutation occurs which quantifies the relationship between altruism and niche founding.

While this analytic investigation of the Wright-Fisher model allows us to consider a single population and its discovery of one other niche, to provide a more comprehensive demonstration of the interaction between altruism and evolutionary exploration, the analytic model is also supplemented in this paper with a simulation of the discovery of new niches from existing ones, similarly to Lehman and Stanley [34]. In the simulation, each niche evolves according to its own Wright-Fisher process as described above, with its own carrying capacity based on the fraction of altruists that it contains. For simplicity we assume that the baseline carrying carrying capacity *N*_base_ is the same for all niches and that the carrying capacity of each niche is independent of the others (i.e., no niche competes with any other). This independence is an important distinction from group selection models in which altruism gives groups a better chance of acquiring fixed resources, as in Traulsen and Nowak’s model [8]. The simulation begins with a single niche composed entirely of nonaltruists. At each generation, every individual has a small probability *μ*_ev_ of undergoing a mutation that founds a new niche. Because this probability is the same for both altruists and nonaltruists, the model provides only an indirect benefit to altruism. That is, altruists do not have any greater chance of founding a new niche, and pay a selective penalty *s*. However, groups containing many altruists benefit from a greater overall probability of mutation.

A characterization of the model’s behavior is obtained for a wide range of parameter settings, varying *N*_mid_ *s* (strength of selection) by three orders of magnitude. All simulations use the settings *N*_base_ = 100 and *μ* = .001. For each combination of parameters, 1,500 simulation runs are performed. S1 Text provides additional details on the simulation.

## Results

This section presents results from both the analytic model and computational simulations, thereby providing a comprehensive view of the effect of altruism when it increases carrying capacity to different degrees.

### Single-niche mathematical model

**Fig 1:**
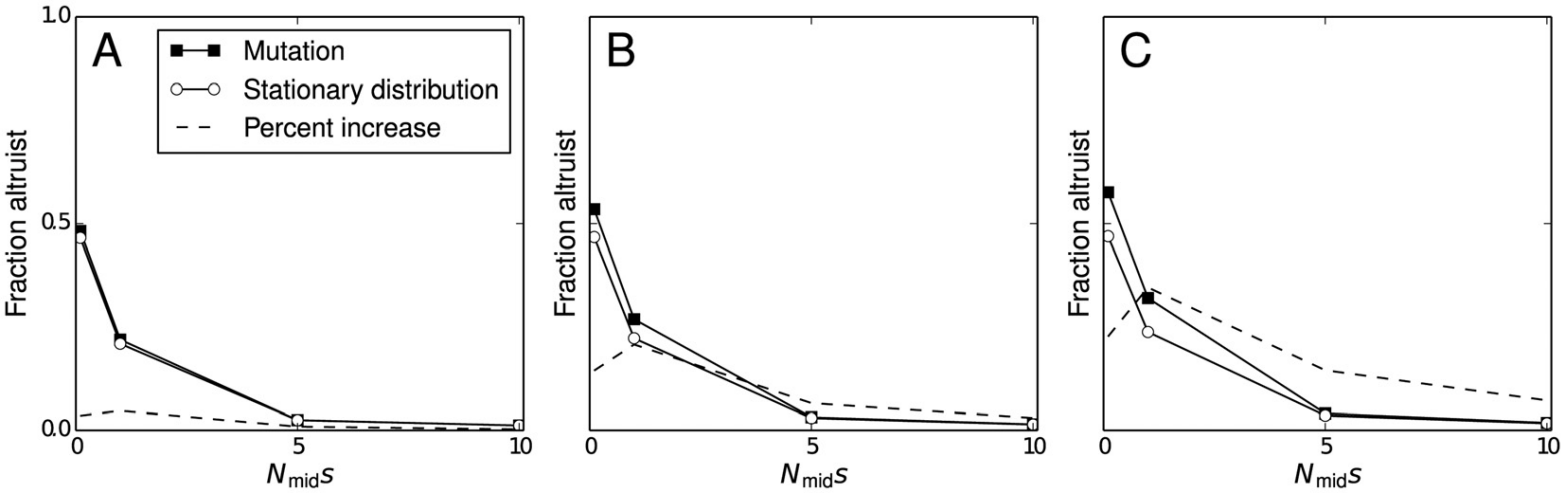
Increase in expected fraction of altruists when a new niche is founded. The open circles give the expectation of the stationary distribution and the closed squares give the expectation conditioned on the occurrence of a niche-founding mutation event. The dashed line shows the percentage increase in the expectation. A) *β* = 1.1. B) *β* = 1.5. C) *β* = 2. All plots use *μ* = .001 and *μ*_ev_ = 5 ⋅ 10^−5^. Results are not significantly different for lower values of *μ*_ev_ (see supplemental information).

To demonstrate the effect of niche-founding mutations, we determine the expectation of the fraction of altruists in the stationary distribution of the model first in general and then conditioned on the occurrence of a mutation event. Because altruists increase the size of the population, any given mutation is more likely to happen when there is a large fraction of altruistic individuals present. Confirming this notion, Fig 1 compares the expected fraction of altruists in the stationary distribution to the expected fraction when a niche-founding mutation event occurs. The expectation conditioned on a mutation event is found using Baye’s theorem and the previously derived stationary distribution. This analysis shows that even when there is strong selective pressure against altruism, a greater fraction of the population is expected to be altruistic when the new niche is discovered. These results are not sensitive to the mutation rate (see S1 Fig). An additional conclusion from this analysis is that the proportion of altruists in the population is robust to the invasion of non-altruists. By the Perron-Frobenius theorem, the eigenvalue of the transition matrix that represents the stationary distribution is unique. That is, the values shown in Fig 1 are the *only* equilibrium of the population dynamics. After an invasion of nonalruists, the population returns to this distribution almost surely after a sufficient number of generations.

### Computational simulation

**Fig 2:**
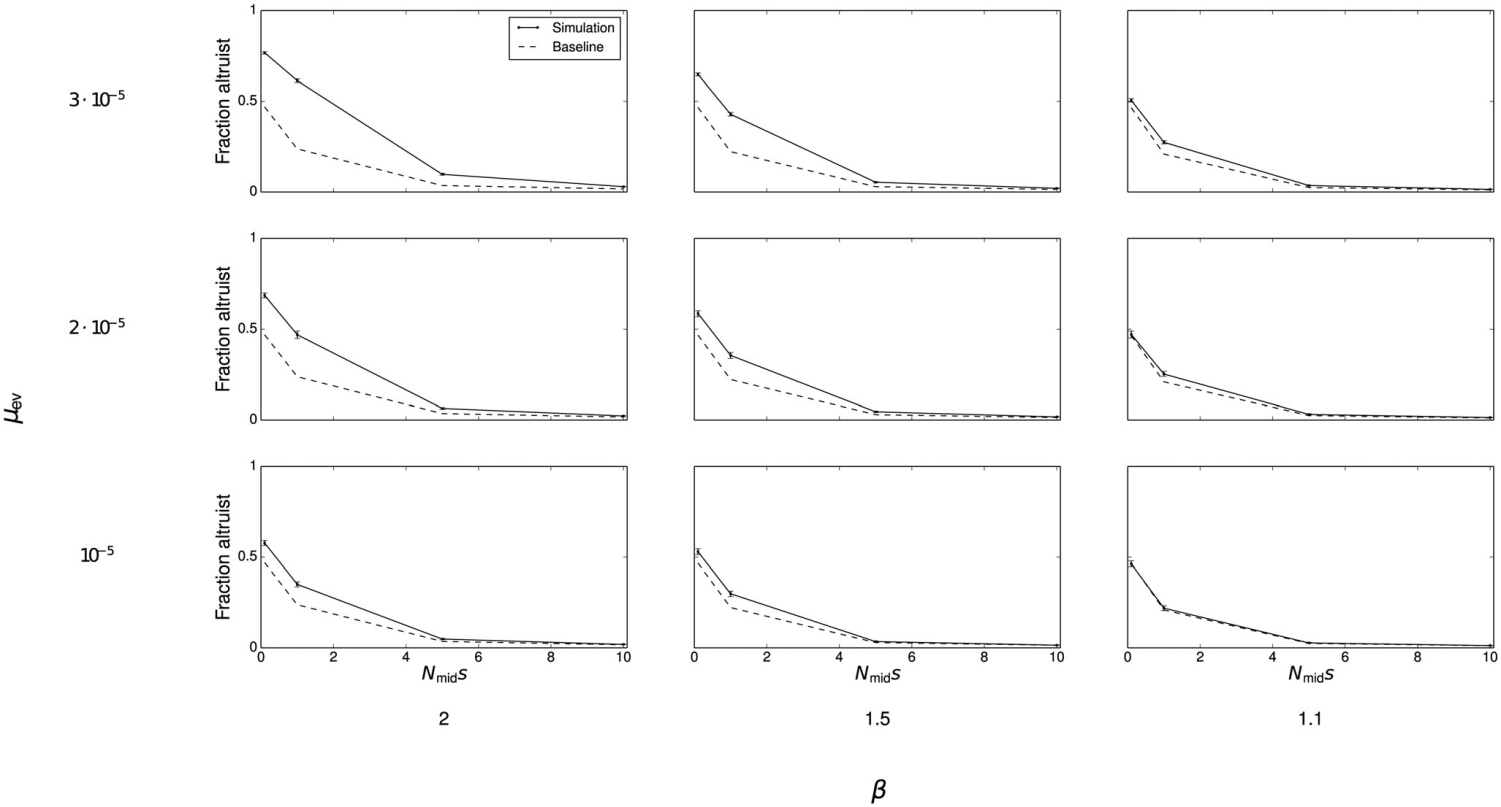
Fraction of altruists in the population after 2,000 generations. Simulation results are given by the solid lines. Dotted lines represent the expectation of the stationary distribution derived analytically for a single niche. Error bars are bootstrapped 99% confidence intervals.

**Fig 3:**
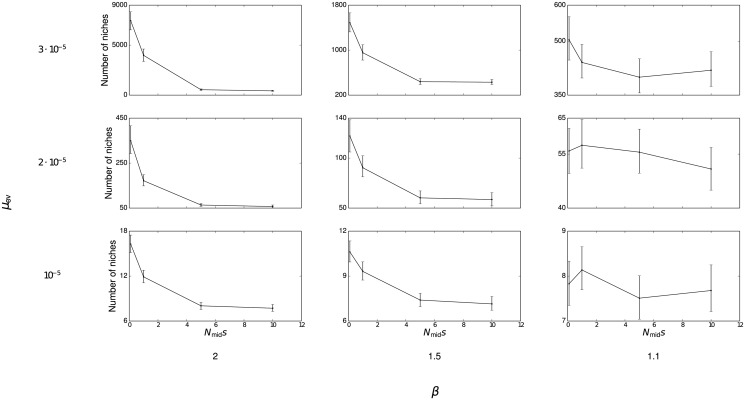
Number of niches occupied after 2,000 generations. Error bars are bootstrapped 99% confidence intervals.

While the analytic model of the previous section demonstrates the effect of altruism on a single new niche, the simulation in this section makes it possible to follow the long-term dynamics as new niches are continually founded. The simulation is run for 2,000 generations, after which the mean fraction of all individuals that are altruists is recorded. Fig 2 shows this fraction as selection strength (*N*_mid_ *s*), the impact of altruist on carrying capacity (*β*) and the probability of founding a new niche (*μ*_ev_) are varied. The results presented here encompass three values of *μ*_ev_ that represent distinct ecological regimes. The first (*μ*_ev_ = 10^−5^) corresponds to a resource-poor environment with only 10–20 available niches. The second (*μ*_ev_ = 2 ⋅ 10^−5^) corresponds to an intermediate environment with a few hundred possible niches. The last (*μ*_ev_ = 3 ⋅ 10^−5^) represents a rich environment that can support thousands of niches. Fig 3 shows the average number of niches present at the end of the simulation under different combinations of parameters. Together, these different cases show that the simulation results hold under a variety of possible ecological conditions.

**Fig 4:**
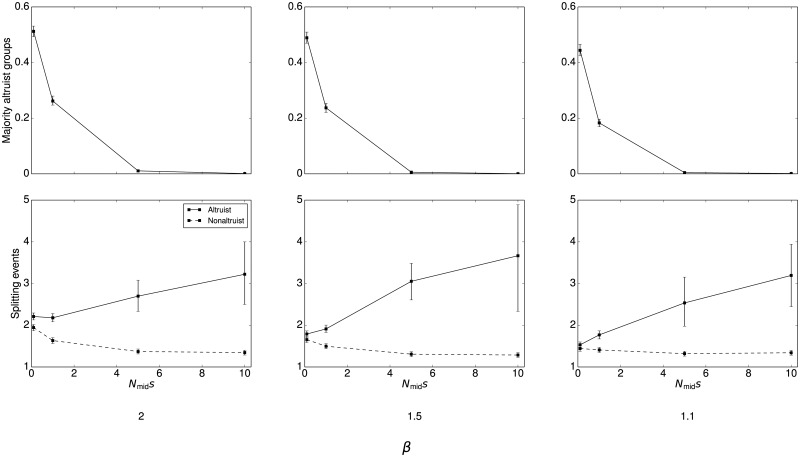
Presence of majority altruistic groups with rare splitting events at generation 2,000. All plots use *μ*_ev_ = 10^−5^. The top row shows that even when selection operates strongly against altruism, a consistent proportion of niches contain mostly altruists. The bottom row shows the number of splitting events needed to produce such niches, compared to those with a majority of nonaltruists. The results show that altruism is produced by the splitting dynamic because majority altruist niches are typically the product of a significantly greater number of splits. Error bars are bootstrapped 99% confidence intervals.

When within-niche selection against altruism is not too severe and *μ*_ev_ takes higher values, a significant fraction, or even majority, of the population can be composed of altruists despite adverse selection. This phenomenon can be attributed to a founder effect: because groups are larger when they have a greater fraction of altruists, altruists are disproportionately likely to found new niches. These individuals reproduce rapidly as they fill the new niche, creating populations that are much more altruistic than would otherwise be expected. In some cases, Fig 2 actually understates the true fraction of altruists (see S2, S3, and S4 Figs for complete time series plots). When splitting events are rare, or selection operates strongly against altruism, altruists comprise only a small proportion of the overall population (Fig 4). However, there are a nonnegligible number of niches with a majority of altruists even when selection against altruism is high. The 99% confidence intervals shown on the figure demonstrate that such groups are not a statistical fluke but rather appear consistently across all runs. Thus, even if selection operates strongly against altruism, and altruistic behavior only minimally increases carrying capacity, we should consistently expect to observe a number of niches in which altruism is common.

Interestingly, because these results hold when selection operates against altruists, it may not be necessary at all to invoke typical benefits of altruism to explain its emergence. However, mechanisms such as inclusive fitness, reciprocal altruism, or intrademic group selection could still operate simultaneously with the proposed effect. Nevertheless, even then, if selection for altruism is relatively weak, niche-founding effects dominate even such positive selective effects.

**Fig 5:**
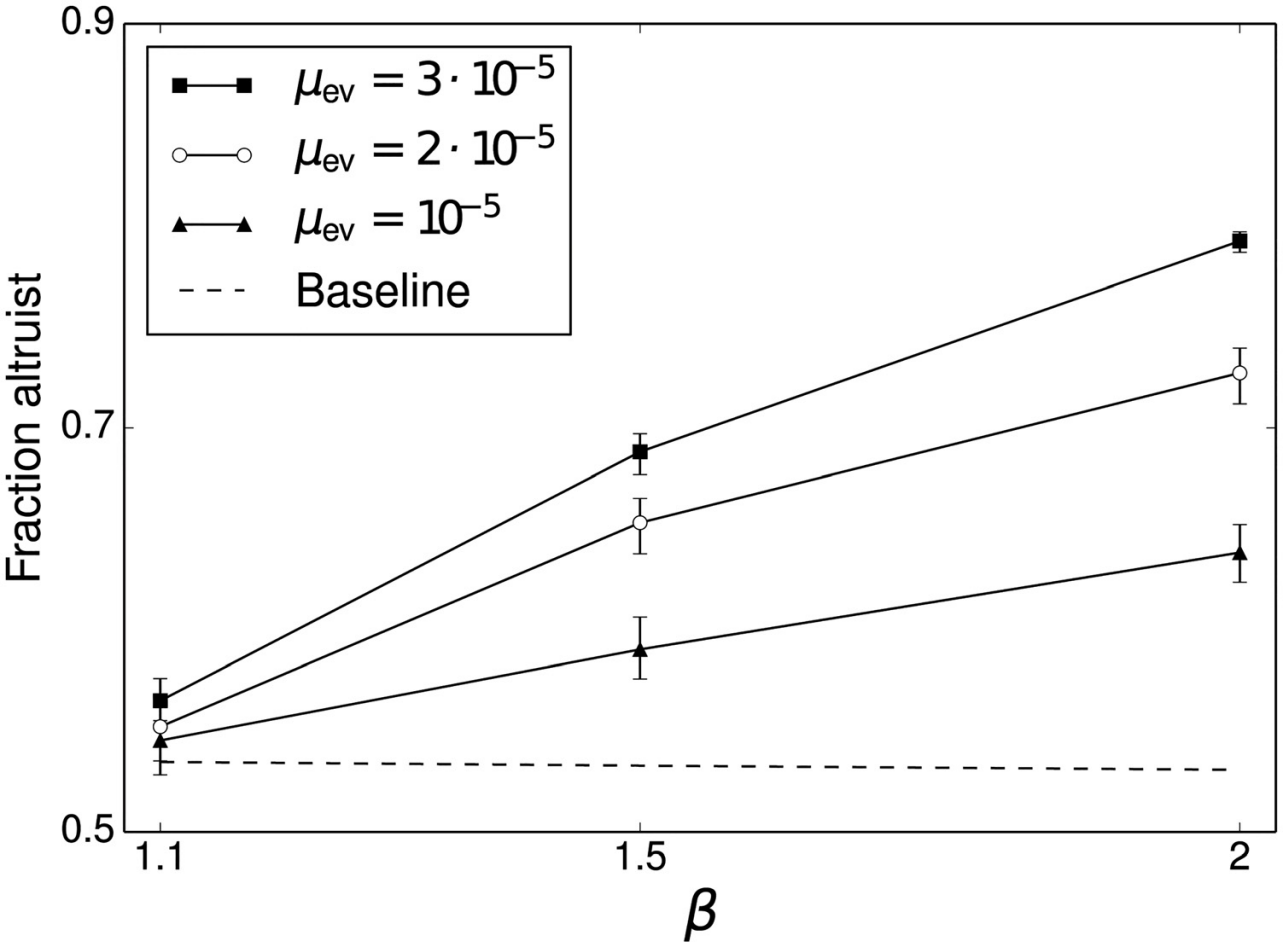
Fraction of altruists with weak selection for altruism. The closed triangles give the mean fraction of altruists with *μ*_ev_ = 10^−5^, the open circles represent *μ*_ev_ = 2 ⋅ 10^−5^, and the closed squares represent *μ*_ev_ = 3 ⋅ 10^−5^. The dashed line gives the expectation of the stationary distribution of a single niche for reference. Simulation results are shown after 2,000 generations with *N*_mid_ *s* = −0.1.

To quantify the impact of positive selection in relation to the niche-founding explanation, the next set of results concerns simulations with a negative value of *N*_mid_ *s*, which means that within-niche selection operates in the opposite direction, in favor of altruism. This scenario represents a generic model of other theories: regardless of the underlying mechanism, their impact can be represented by assuming that the evolutionary dynamics of a given niche favor altruism. Fig 5 shows the ending fraction of altruists under *N*_mid_ *s* = −0.1, representing weak selection in favor of altruism. Even under these conditions, the impact of evolutionary exploration is notable. The baseline evolutionary dynamics support a mean fraction of slightly over fifty percent altruists, but the population achieves a significantly higher level under the simulated multi-niche process. When selection operates strongly in favor of altruism, virtually the entire population is composed of altruists in the single niche case, so little significant impact is possible (see S4, S5, and S6 Figs for complete time series plots). Thus these results are most relevant to the case where previously proposed mechanisms exert a weak effect that would not be significant enough on its own to ensure that the population is mostly composed of altruists. In other words, the niche-founding effect dominates the weak effect in the scenario of conventional explanations.

## Discussion

The implications of these findings are important for evolutionary theories of altruism. Because altruism supports individuals who would not otherwise survive, it facilitates evolutionary exploration of paths that would not otherwise have been possible. Therefore, at times when a large diversity of species is observed, it is reasonable to expect to see altruism as an enabling condition. Because evolution is a stochastic process, even when selection is strongly against altruism it should still arise at times by chance. These are the very times that would be most favorable to the founding of new niches.

New niches, in turn, benefit altruistic individuals. Because populations are disproportionately altruistic at the times when such niches are discovered, the new niches are more likely to be founded by altruists than would otherwise be expected. Thus the advantages of filling a previously undiscovered niche accrue more often to altruists, which helps increase the prevalence of altruistic behavior even in the face of selective disadvantages. This virtuous cycle drives the emergence of altruism across a wide range of selective environments within the individual niches. Essentially, group selection can operate over the space of niches through increased exploration, instead of through competition against other groups.

A similar idea is explored by Morgan et al. [35], who show that altruism can be sustained in populations of the bacterium *Pseudomonas fluorescens* because larger altruist populations generate greater numbers of beneficial mutations. While not dealing specifically with the creation of new environmental niches, as is proposed here, this study lends empirical credence to the idea that raising the number of mutations can sustain altruistic populations against adverse selection.

Perhaps even more interesting is the result obtained for the case where there is strong selective pressure against altruism within each niche. Here, the model shows that we should still consistently expect to see a noticeable fraction of species with high levels of altruism. In fact, it could be possible to witness empirical examples of altruistic species even if mechanisms such as conventional group selection or inclusive fitness fail. This surprising insight provides a new framework in which to consider the evolution of altruism. It cannot be taken for granted that altruistic behavior helps altruists compete against others in the population. Traditional explanations for altruism uniformly deal with the need for altruists to compete for limited space in the niche by securing an advantage for themselves or their relatives. However, when a fuller evolutionary process is considered, such explanations may not be necessary. While it is important to note that this insight does not provide any evidence against specific theories for the evolution of altruism, it implies that before such theories are invoked it must be demonstrated that they are needed at all. In effect, our null model should include the presence of some of altruistic species.

A further implication is that if mechanisms that select for altruism within the niche are in fact present, then only weaker claims need to be made in their favor because the population is predisposed to higher levels of altruism than would be expected under a typical neutral model. Previous research has shown in a number of contexts that the benefit to cost ratio of altruism must exceed some threshold, which is specific to the theory being proposed. One prominent example is Hamilton’s coefficient of relation [2], *r* > *c*/*b*, which states that for altruism to spread under inclusive fitness, altruists’ relatedness to those that they assist must exceed the cost-benefit ratio to altruism. Another result is derived by Traulsen and Nowak [8] in the context of multilevel selection: *b*/*c* > 1+(*n*/*m*). Here, the benefit to cost ratio for altruism must exceed 1 plus the ratio of the size of a group to the number of groups present. However, results here show that such thresholds might not be so stringent if the proposed innovation effect biases populations towards higher levels of altruism from the start. That is, only weaker benefits for altruism would be necessary for such mechanisms to operate. An interesting direction for future work is to further explore the relationship between different kinds of group selection. It could be possible to place the present results within the same mathematical framework as previous theories, just as a number of group selection and inclusive fitness models have been formally unified [36–38].

## Conclusion

This work has proposed a novel form of group selection that depends only on the ecological structure of niches, not selection for altruism within any given population. An extension of the Wright-Fisher model as well as computational simulation shows that when altruists help support other individuals, new niches are more likely to be discovered. This dynamic drives the spread of altruism through further populations. While virtually all previous work on the topic assumes that there must be some mechanism which helps altruistic individuals or groups compete against others in the population, the surprising conclusion is that no such force may be necessary. Future work can more tightly integrate the present results with other theories and consider exactly how the process of niche discovery changes the requirements for altruism to evolve.

Furthermore, this work has focused on the impact of increasing the overall number of individuals within a niche without focusing on individual differences. Altruism could reduce the pressure of selection by supporting unique individuals whose phenotypes would not ordinarily allow them to survive. Such genetic paths would otherwise be unexplored, so flattening the pressure of selection might in turn further increase the diversity of new niches that are found. Future work can also investigate this possibility.

Placing altruism in the broader context of evolutionary innovation can help to illuminate how such paradoxical behaviors come to exist. Altruists may not compete more effectively against others within the niche but instead help to fuel the incredible diversity that evolution has produced.

## Supporting Information

S1 Text Mathematical Model and Simulation Details. (PDF)

S1 Fig Expected fraction of altruists in the stationary distribution of the Markov process with low mutation rate. Here, *μ*_ev_ = 1 ⋅ 10^−5^. The values are nearly identical to those with *μ*_*ev*_ = 5 ⋅ 10^−5^ shown in Fig 1 of the main text. The open circles give the expectation of the stationary distribution and the closed squares give the expectation conditioned on the occurrence of a mutation event. The dashed line shows the percentage increase in the expectation. A) *β* = 1.1. B) *β* = 1.5. C) *β* = 2. All plots use *μ* = .001, *μ*_ev_ = 1 ⋅ 10^−5^, and *N*_base_ = 100.
(EPS)

S2 Fig Mean fraction of altruists at each generation with selection against altruism and high mutation rate. The shaded region gives bootstrapped 99% confidence intervals. Dotted lines represent the expectation of the stationary distribution derived analytically for a single niche. In some cases, an asymptote has been reached. In others, simulation to an asymptote is computationally infeasible, but in these cases the trend is uniformly increasing. This outcome demonstrates that results in the main text, which use the mean fraction of altruists after 2,000 generations, can only understate the true fraction of altruists expected as time increases. These plots use *μ*_ev_ = 3 ⋅ 10^−5^.
(EPS)

S3 Fig Mean fraction of altruists at each generation with selection against altruism and medium mutation rate. The shaded region gives bootstrapped 99% confidence intervals. Dotted lines represent the expectation of the stationary distribution derived analytically for a single niche. In most cases, an asymptote has been reached. These plots use *μ*_ev_ = 2 ⋅ 10^−5^.
(EPS)

S4 Fig Mean fraction of altruists at each generation with selection against altruism and low mutation rate. The shaded region gives bootstrapped 99% confidence intervals. Dotted lines represent the expectation of the stationary distribution derived analytically for a single niche. In most cases, an asymptote has been reached. These plots use *μ*_ev_ = 1 ⋅ 10^−5^.
(EPS)

S5 Fig Mean fraction of altruists at each generation with selection for altruism and high mutation rate. Here, *μ*_ev_ = 3 ⋅ 10^−5^. For higher values of *β*, the simulation becomes computationally infeasible due to the expontential rise in the number of groups. The shaded region gives bootstrapped 99% confidence intervals. As referenced in the main text, when selection for altruism is strong, there is no room for any additional effect because virtually the entire population is composed of altruists due to selection alone.
(EPS)

S6 Fig Mean fraction of altruists at each generation with selection for altruism and medium mutation rate. Here, *μ*_ev_ = 2 ⋅ 10^−5^. The shaded region gives bootstrapped 99% confidence intervals. As referenced in the main text, when selection for altruism is strong, there is no room for any additional effect because virtually the entire population is composed of altruists due to selection alone.
(EPS)

S7 Fig Mean fraction of altruists at each generation with selection for altruism and low mutation rate. Here, *μ*_ev_ = 1 ⋅ 10^−5^. The shaded region gives bootstrapped 99% confidence intervals. As referenced in the main text, when selection for altruism is strong, there is no room for any additional effect because virtually the entire population is composed of altruists due to selection alone.
(EPS)
