# Supplementary material for: Altruists Proliferate Even at a Selective Disadvantage within Their Own Niche
Source: PLoS One. 2015 Jun 1;10(6):e0128654. doi: 10.1371/journal.pone.0128654 (PMC4451246; doi:10.1371/journal.pone.0128654)
Supplement: S1 Text — (PDF) [file pone.0128654.s001.pdf]

Supplementary Material: Altruists Proliferate Even at a Selective Disadvantage within Their Own Niche

Bryan Wilder[1], Kenneth O. Stanley[1],

1 Department of Electrical Engineering and Computer Science, University of Central Florida, Orlando, FL, USA ∗ E-mail: bryan.wilder@knights.ucf.edu

Analytic model

The Wright-Fisher model is the classical population-genetic representation of generational evolution in a finite population. Here, the population is of fixed size N, and individuals carry one of two alleles A_1 and A_2. The allele A_2 confers some excess fitness s relative to A_1. The frequency of the two alleles in the population is given by a Markov process where the states are represented by the number of A_2 individuals. At each generation, individuals are chosen uniformly with replacement to reproduce. There is a probability μ of a mutation occurring for each reproductive event (the mutation probabilities are assumed equal for the two alleles). Therefore, the probability of transitioning from a state with i individuals of type A_2 to one with j is

$$p_{i \to j} = \binom{N}{j} (p_{A_2})^j \left(1 - p_{A_2}\right)^{N-j}, \tag{1}$$

where the probability p_{A_2} of selecting an A_2 individual is

$$p_{A_2} = (1-\mu)\frac{(1+s)i}{(1+s)i + (N-i)} + \mu\frac{N-i}{(1+s)i + (N-i)}. \tag{2}$$

We generalize the Wright-Fisher model to allow the population to vary according to the proportion of altruists that it contains. In this model, the altruistic allele is represented by A and the selfish (non-altruistic) allele is represented by S. The states of the Markov process are then given by the number of both A and S individuals. If there are i altruists and j non-altruists, then the carrying capacity for the next generation is

$$N_{t+1} = \left\lfloor N + N(\beta - 1)\frac{i}{i+j} \right\rfloor. \tag{3}$$

Thus N_{t+1} varies linearly between N when the population is entirely non-altruists and βN when the population is entirely altruists. Here, $\beta > 1$ is a parameter that controls the strength with which altruism increases carrying

capacity. The floor is taken because only an integer number of individuals can be selected. Thus the transition probability $p_{ij\to kl}$ of going from i individuals of type A and j individuals of type S to k and l respectively is

$$p_{ij\to kl} = \binom{N_{t+1}}{l} (p_S)^l (1-p_S)^{N_{t+1}-l}, \tag{4}$$

where p_S is given by

$$p_S = (1-\mu)\frac{(1+s)j}{i+(1+s)j} + \mu\frac{i}{i+(1+s)j}. \tag{5}$$

Because $\mu > 0$, each state can be reached from any other state in a finite number of steps, making the Markov chain ergodic. Accordingly, it has a stationary distribution π that is reached independently of initial condition as $t \to \infty$. The transition matrix combined with the condition that for all states (i,j), $\sum_{(k,l)} p_{ij\to kl} = 1$ gives a system of linear equations that, when solved, yields the probabilities for each state in the stationary distribution.

Once π has been computed, we can consider the probability of observing a given fraction of altruists in the population when a mutation event occurs that discovers a new niche, assuming that enough generations have passed for π to give the probabilities of each state. Suppose that at each generation, every new individual has a probability μ_{ev} of mutation to a new niche. Let $\mathtt{m}$ denote the occurrence of a mutation event and $x = (i,j)$ denote that the Markov chain is in state (i,j) Then, by Baye's Theorem, the posterior probability of being in state (i,j) when such a mutation event occurs is

$$p[x=(i,j)|\mathtt{m}] = \frac{p[\mathtt{m}|x=(i,j)]p[x=(i,j)]}{p[\mathtt{m}|x=(i,j)]p[x=(i,j)] + p[\mathtt{m}|x\neq(i,j)]p[x\neq(i,j)]} \tag{6}$$

with

$$p[\mathtt{m}|x=(i,j)] = \mu_{\text{ev}}(i+j) \tag{7}$$

and

$$p[\mathtt{m}|x\neq(i,j)] = \sum_{(k,l)\neq(i,j)} \mu_{\text{ev}}(k+l)p[x=(k,l)|x\neq(i,j)]. \tag{8}$$

By comparing the prior probability of a state in the stationary distribution to its posterior probability given that a mutation event is occurred, we can thereby quantify the overrepresentation of altruists in conditions of evolutionary exploration.

Time series simulation details

Although the number of groups that can appear over the course of a run is unbounded in principle, due to practical constraints on computation, simulations are limited to 2^{15} niches, restarting runs that cross this threshold. However, as shown in the results in the main text and the previous section, it is important to note that any bias introduced by this constraint should weigh *against* the development of altruism because simulations with more groups tend to have more altruists.

As noted in the main text, 100 simulations are performed for $\mu_{\text{ev}} = 5 \cdot 10^{-5}$, while 1,500 are performed for $\mu_{\text{ev}} = 10^{-5}$. This difference reflects that runs for $\mu_{\text{ev}} = 10^{-5}$ are significantly more efficient, but also significantly more variable. However, bootstrapped confidence intervals show that the degree of precision achieved is comparable for both cases, highlighting the justification behind these choices.
